# Genome-Wide Identification of the Peanut *ASR* Gene Family and Its Expression Analysis under Abiotic Stress

**DOI:** 10.3390/ijms252011008

**Published:** 2024-10-13

**Authors:** Jiaxing Li, Mingxia Ma, Tuo Zeng, Lei Gu, Bin Zhu, Hongcheng Wang, Xuye Du, Xiu Zhu

**Affiliations:** 1 School of Life Sciences, Guizhou Normal University, Guiyang 550003, China; 222100100426@gznu.edu.cn (J.L.); zengtuo@gznu.edu.cn (T.Z.); leigu1216@nwafu.edu.cn (L.G.); 201703008@gznu.edu.cn (B.Z.); wanghc@gznu.edu.cn (H.W.); 2 Guizhou Academy of Testing and Analysis, Guizhou Academy of Sciences, Guiyang 550003, China; mamingxia@gzata.cn

**Keywords:** peanut, *AhASR*, abiotic stress, expression analysis

## Abstract

Peanut (*Arachis hypogaea* L.) is one of the most important oil and food legume crops worldwide. *ASR* (abscisic acid, stress, ripening) plays extremely important roles in plant growth and development, fruit ripening, pollen development, and stress. Here, six *ASR* genes were identified in peanut. Structural and conserved motif analyses were performed to identify common ABA/WDS structural domains. The vast majority of *ASR* genes encoded acidic proteins, all of which are hydrophilic proteins and localized on mitochondria and nucleus, respectively. The cis-element analysis revealed that some cis-regulatory elements were related to peanut growth and development, hormone, and stress response. Under normal growth conditions, *AhASR4* and *AhASR5* were expressed in all tissues of peanut plants. Quantitative real-time PCR (qRT-PCR) results indicated that peanut *ASR* genes exhibited complex expression patterns in response to abiotic stress. Notably, under drought and cadmium (Cd) stress, the expression levels of *AhASR4* and *AhASR5* were significantly upregulated, suggesting that these genes may play a crucial role in the peanut plant’s resistance to such stressors. These results provide a theoretical basis for studying the evolution, expression, and function of the peanut *ASR* gene family and will provide valuable information in the identification and screening of genes for peanut stress tolerance breeding.

## 1. Introduction

Plants may be affected by abiotic stressors, such as heavy metals, high salt, drought, and low temperature, during growth and development, however, plants cope with abiotic stress through different adaptive mechanisms, such as altered growth morphology, physiology and biochemistry, and gene expression trends [1]. ASR proteins are a group of proteins that are uniquely expressed during plant maturation and are able to be expressed in various plant organs in response to stimuli, such as ABA and adversity [2]. They play an important role in plant response to adversities, such as drought, high salt, low temperature, and abscisic acid, and are important regulators of plant growth and development, fruit ripening, and sugar metabolism [3]. ASR proteins are characterized by their high hydrophilicity, low molecular weight, and specificity for binding to plant tissue DNA [4]. ASR is rich in amino acids, such as alanine, glutamic acid, histidine, and lysine, which are predominantly basic amino acids [5]. Known ASR proteins have been shown to have a zinc-binding structural domain at the N-terminus and a putative nuclear targeting signal at the C-terminus [6].

The *ASR* gene was initially discovered in tomatoes [7]. Subsequent studies demonstrated that ASR is localized in the nucleus [8], and its presence was later confirmed in both the nucleus and cytoplasm [9]. *ASR* genes are not only implicated in plant responses to drought, high salinity, low temperatures, and abscisic acid stress, but they also play a vital role in various aspects of plant life, including development, maturation, and sugar metabolism [10]. Interestingly, ASR proteins and their homologous sequences may not be present in the model organisms *Arabidopsis thaliana* and Brassicaceae [11,12]. *ASR* genes have been extensively studied in various species, including corn, rice, chickpea, and apple [13,14,15,16]. However, significant gaps remain in the analysis of the peanut *ASR* gene family, particularly concerning its response mechanisms to salt stress, drought stress, aluminum (Al) stress, and Cd stress.

*ASR* genes play a crucial role in the plant’s response to various stressors, including drought, high salinity, heavy metals, and abscisic acid (ABA) [17,18,19,20,21,22]. Research has demonstrated that the upregulation of *SlASR* expression in *Suaeda liaotungensis* can enhance the resistance of transgenic *Arabidopsis thaliana* to salt, drought, and freezing conditions [23]. In tomatoes, the expression of *ASR1* and *ASR4* is upregulated in response to salt and osmotic stress, resulting in a higher survival rate for tomatoes overexpressing *ASR1* under water stress [24]. The *SiASR4* gene improves tolerance in *Arabidopsis thaliana* by regulating stomatal function and activating the transcription of related genes [25]. *OsASR5* enhances Al tolerance by modulating the expression of aluminum stress response genes, while *TaASR1* in wheat strengthens the antioxidant system by regulating associated genes to improve drought tolerance [26,27]. The *ASR* gene in *Tamarix chinensis* increases the activity of antioxidant enzymes, reduces the accumulation of reactive oxygen species (ROS) under salt stress, and enhances salt tolerance [28]. Furthermore, the overexpression of *OsASR1* can enhance salinity and drought tolerance, resulting in improved crop yields [29]. In summary, *ASR* genes significantly bolster plant resistance by modulating the response mechanisms to various stress conditions.

Peanut (*Arachis hypogaea* L.) is an allotetraploid crop recognized for its valuable edible fats and proteins [30]. It ranks among the most significant oilseed and food legume crops globally, contributing substantial value to both the diet and economy of various countries [31,32]. However, in certain peanut-growing regions, drought and salt stress are the primary factors limiting peanut growth and productivity, leading to a significant reduction in agricultural production [33,34,35]. Peanut is classified as an underground fruiting legume. In addition to the absorption capacity of their roots, the pods of peanut plants can also absorb nutrients, which leads to a more pronounced accumulation of heavy metals [36]. The roots of peanut plants exhibit heightened sensitivity to Al, and the differential expression of hormone-related genes under conditions of elevated aluminum concentration can result in various adverse effects, including reduced root biomass and impaired root development [37,38]. Research indicates that peanut is particularly susceptible to Cd contamination. Cd is a toxic heavy metal that poses significant risks to food quality and human health [39,40]. Although the concentrations of Cd and other heavy metals in the soil of most peanut-producing regions in China generally comply with the quality standards established by the Ministry of Agriculture for green food production areas, there are occasional instances in which peanut samples exceed these standard levels [41]. Therefore, it is essential to investigate the impact of heavy metals on peanut.

This study conducted whole genome sequence analysis of peanut and identified six members of the *ASR* gene family. We analyzed the gene structure, conserved motifs, phylogenetic relationships, cis-acting elements, and protein interaction networks of *AhASRs*. Additionally, we examined the expression patterns of these genes in the leaves and roots of peanut under four different abiotic stressors. The results provide a foundation for future in-depth investigations into the biological functions of peanut *ASRs*.

## 2. Results

### 2.1. Identification and Chromosomal Localization of Peanut ASR Genes

**Figure 1:**
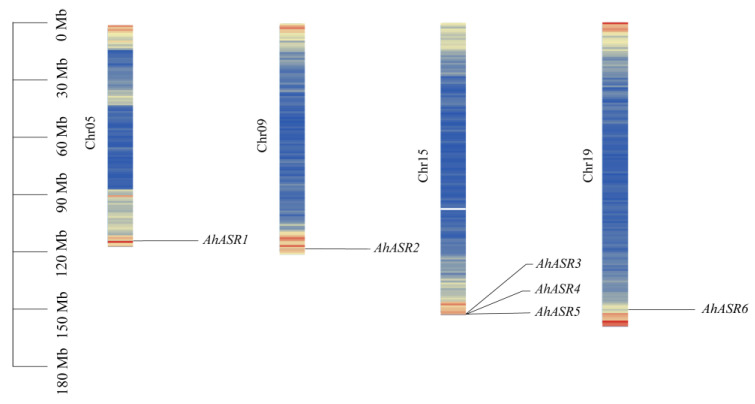
Chromosome distribution of *AhASR* members.

Six peanut *ASR* genes were identified by genome-wide analysis and named *AhASR1–AhASR6* in ascending order of their chromosomal position on the chromosome. The *ASR* genes in peanut were all located near the end of the long arm of the chromosome and were unevenly distributed. *AhASR1* was located on Chr5, and *AhASR2* was located on Chr9. *AhASR3*, *AhASR4*, and *AhASR5* were present in clusters on peanut Chr15, and *AhASR6* was located on Chr19 (Figure 1).

### 2.2. Physicochemical Properties Analysis of ASR Protein Sequences in Peanut

The physicochemical properties analysis of the peanut *ASR* gene family revealed that all 6 members had 2 exons, with open reading frame lengths ranging from 345 to 673 bp, coding amino acid numbers ranging from 114 to 223, molecular mass sizes ranging from 13.18 to 23.96 kDa, and proteins with theoretical isoelectric points below 7, suggesting that the ASR proteins of peanut were abundant in acidic amino acids (Table 1). The instability coefficients of AhASR4 and AhASR5 among the members of AhASRs were both less than 40, suggesting that these proteins were stable. The lipid indices of AhASR proteins ranged from 23.77 to 56.84, indicating that these AhASR proteins were heat-resistant proteins. The negative average hydrophilicity values indicated that all AhASR proteins were hydrophilic. Subcellular localization predictions indicated that AhASR1, AhASR3, AhASR4, and AhASR5 were localized in the mitochondria, and AhASR2 and AhASR6 were localized in the nucleus. The secondary structure of peanut ASR proteins was analyzed by the online website SOPMA. The results showed that peanut ASR proteins were mostly dominated by α-helix, accounting for 30.49–55.56%. Irregular helix was the second largest, accounting for 25.44–49.33%. β-sheet and extended chain were the least, accounting for 5.98–13.45% and 6.73–10.26%, respectively. Upon analyzing the predicted protein tertiary structures of AhASRs, it was observed that AhASR2 and AhASR6 exhibited high similarity and complexity. Similarly, AhASR3, AhASR4, and AhASR5 also showed a high degree of similarity in their tertiary structures. In contrast, AhASR1 displayed a significantly different tertiary structure compared to other ASRs in peanut, suggesting a potential divergence in function from other ASR proteins in the same species (Table S1).

### 2.3. Phylogenetic Relationships among ASR Genes

**Figure 2:**
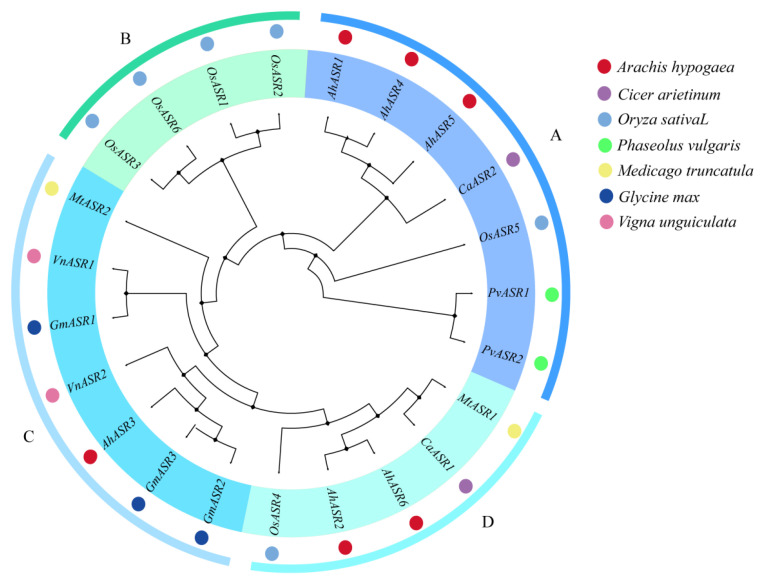
A phylogenetic analysis of ASR proteins from peanut, soybean, cowpea, chickpea, kidney bean, alfalfa, and rice. The various colors and letters of the outer circle represent four distinct branches.

To further understand the evolutionary relationships of peanut ASR proteins. A phylogenetic tree was constructed using the amino acid sequences of AhASRs with 23 ASR amino acid sequences from a total of seven species, including soybean (*Glycine max*), cowpea (*Vigna unguiculata*), chickpea (*Cicer arietinum*), kidney bean (*Phaseolus vulgaris*), alfalfa (*Medicago truncatula*), and rice (*Oryza sativa*). All ASRs were categorized into 4 groups by phylogenetic tree affinities, where Group A, Group B, Group C and Group D had 7, 4, 7, and 5 ASRs, respectively. AhASR1, AhASR4, and AhASR5 in Group A were more closely related to CaASR2. AhASR3 was highly homologous to GmASR2 and GmASR3 in Group C. AhASR2 and AhASR6 were also highly homologous to CaASR2 and MtASR1 in Group D (Figure 2). The ASRs of peanut and chickpea were more closely related, suggesting that their structures and functions may show greater similarity.

### 2.4. Structural and Conserved Motif Analysis of ASR Genes in Peanut

**Figure 3:**
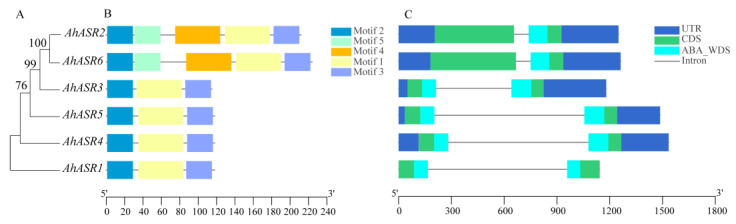
Phylogenetic, structural, and conserved motif analysis of *ASR* genes in peanut. (**A**) Phylogenetic tree of AhASRs. (**B**) Motif analysis of AhASRs. Colored boxes indicate the different conserved motifs as indicated in the scheme to the right of the figure. (**C**) Exon–intron structures of *AhASRs*.

To analyze the role of *ASRs* in depth, the peanut *ASR* gene structures and protein conserved motifs were analyzed (Figure 3). A total of five conserved motifs were obtained in AhASR proteins. Among them, motif 1, motif 2, and motif 3 were highly conserved and present in all AhASR proteins. It is hypothesized that they may serve specific functions in peanut. In terms of gene structure, all *AhASR* genes had UTRs except *AhASR1*, and all *ASR* genes had two introns and two exons. Initial speculation was that during evolution, the introns of *ASR* genes became shorter and shorter as they evolved to withstand adversity.

### 2.5. Sequence Analysis of AhASR Amino Acids

**Figure 4:**
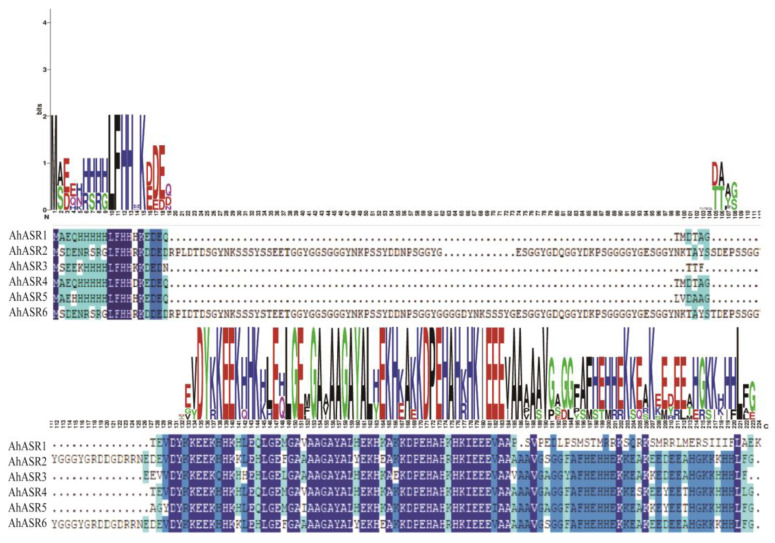
Alignments of AhASR amino acid sequences.

To gain a deeper understanding of the differences among the AhASR proteins, multiple alignment analysis revealed that the amino acid sequences of the AhASRs were highly consistent and conserved. There were two highly conserved regions in the peanut ASR protein sequence: a histidine-rich region at the N-terminal end and a C-terminal end with an ABA/WDS structural domain and the putative nuclear targeting signal (Figure 4).

### 2.6. Cis-Acting Elements Analysis of AhASRs

**Figure 5:**
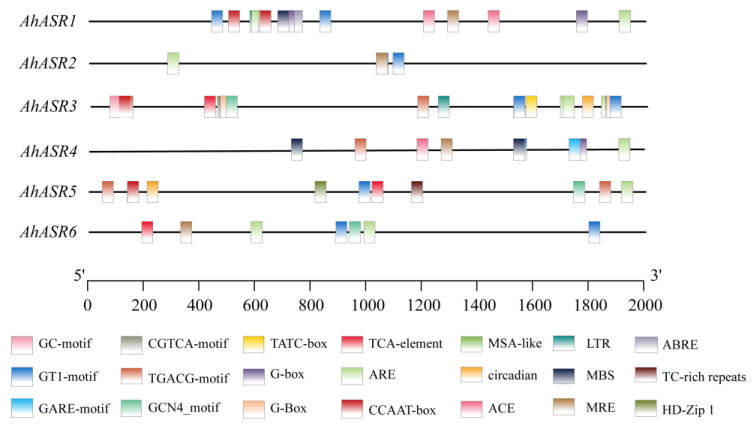
Distribution of cis-acting elements in the promoter region. Graphs in different colors represent different classes of cis-acting elements.

To understand the function of the peanut *ASR* gene, cis-acting element analysis was conducted on the upstream 2000-bp nucleotide sequence of the *AhASR* transcription start site (Figure 5). The promoter region of the *AhASR* genes was characterized by the presence of various cis-acting elements, including endosperm-specific expression elements, light-responsive elements, circadian rhythm control elements, and low-temperature-responsive elements. Additionally, it contained salicylic acid response elements, ABA response elements, MeJA response elements, GA response elements, defense stress elements, hormone signaling response elements, and MYB binding sites associated with drought responses induced by abiotic stress. This suggests that *AhASRs* may play a role in regulating peanut plant growth and various response processes. It is important to note that not all *AhASRs* contained all cis-acting elements. For instance, the drought responsive element MYBs were found only in *AhASR1* and *AhASR4*, and the low-temperature responsive element LTR was exclusive to *AhASR1* and *AhASR3*. *AhASR3* had the least cis-acting elements with only the anaerobic inducer ARE and the photoresponse element MRE, GT1 motif. These findings suggest that the peanut *ASR* gene family has undergone or is undergoing functional differentiation during evolution.

### 2.7. Gene Duplication and Inter-Species Collinearity Analysis of AhASRs

**Figure 6:**
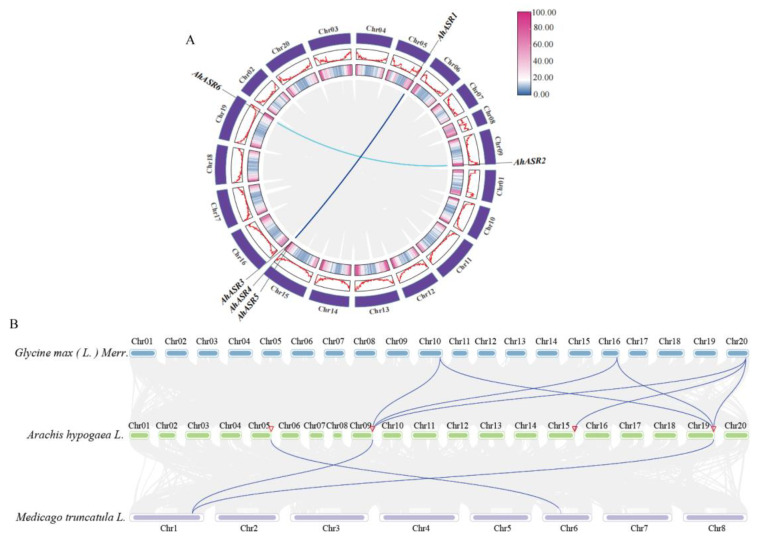
Gene duplication and collinearity analysis of AhASRs (**A**) Inter-species collinearity analysis of *ASR* genes of peanut, soybean, and alfalfa. (**B**) The lines in blue represent collinearity relationships among the ASR genes. The two outermost circles in Fig. A represent gene density.

The replication events in *AhASR* genes were analyzed using MCScanX. The results revealed that six *AhASR* genes formed two pairs of segmental duplications: *AhASR1* and *AhASR4* as well as *AhASR2* and *AhASR6* (Figure 6A). Additionally, a tandem duplication of *AhASR4* and *AhASR5* was observed on Chr15. These findings suggest that the expansion of the peanut *ASR* gene family may be attributed to both segmental and tandem duplication events. The Ka/Ks ratios of the two collinear pairs of *AhASR*, calculated using TBtools, were found to be 0.3067 and 0.0275, respectively. This indicates that the *AhASR* gene family has primarily been influenced by purifying selection pressure during its evolution. The divergence times of homologous genes were estimated to be 15.8303 and 5.09468 million years ago (Mya) (Table S3).

The evolutionary relationships of the *AhASR* gene family were investigated by analyzing the collinearity of *ASR* genes among peanut, soybean, and alfalfa. The results identified seven pairs of collinear *ASR* genes between soybean and peanut as well as three pairs between alfalfa and peanut (Figure 6B). These results suggest a greater degree of homology between the *ASR* gene families of soybean and peanut compared to that of alfalfa. Furthermore, we evaluated the Ka/Ks values of the seven pairs of collinear genes in peanut and soybean (Table S4). The findings indicate that all pairs of collinear genes exhibit Ka/Ks ratios of less than 1, implying that the *AhASR* gene family has evolved under purifying selection.

### 2.8. Analysis of the Protein–Protein Interaction Network of AhASR Family Genes

**Figure 7:**
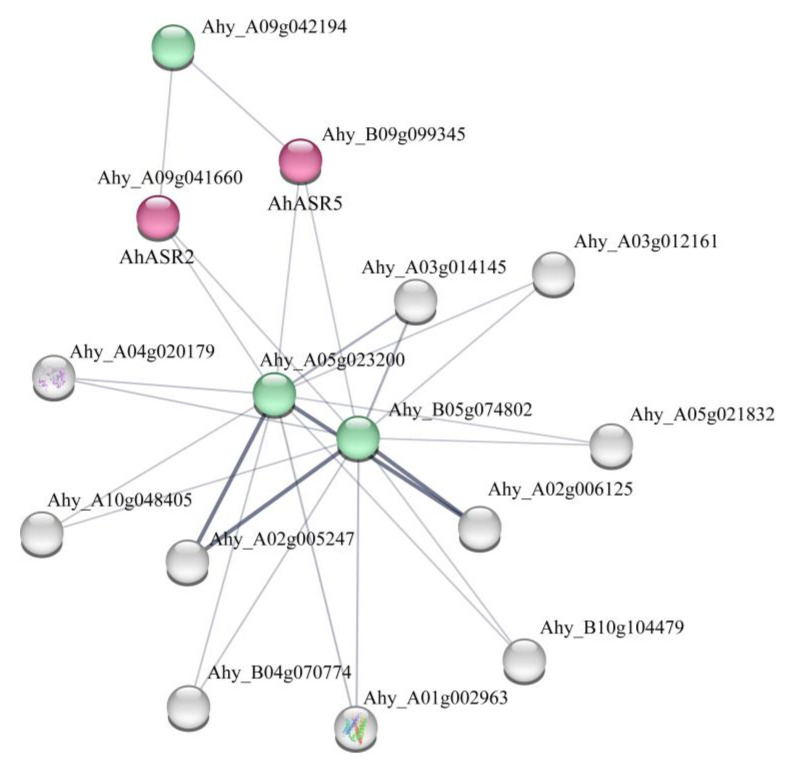
Interaction network analysis of AhASR proteins. The STRING database was employed to predict the protein–protein interaction networks. The red color represents the AhASR proteins, while the green color indicates other proteins that interact directly with AhASRs.

To enhance our understanding of the role of ASR genes in peanut, we utilized the STRING database to construct a potential protein–protein interaction (PPI) network, which included AhASR proteins and other proteins encoded in the peanut genome (Figure 7, Table S5). The PPI network comprised 17 nodes and 26 edges, with 3 proteins identified as interacting with the AhASR2 and AhASR5 proteins in peanut. These three interacting proteins included one member containing a C2H2-type domain (Ahy_A09g042194) and two members of the peroxidase reductase family (Ahy_B05g074802 and Ahy_A05g023200). Notably, these two peroxidase redox proteins (Ahy_B05g074802 and Ahy_A05g023200) are crucial for the detoxification of superoxide and the protection of cells against oxidative stress. Therefore, we predict that peanut may interact with these two proteins and participate in this biological process.

### 2.9. Expression Pattern Analysis of AhASR Genes

**Figure 8:**
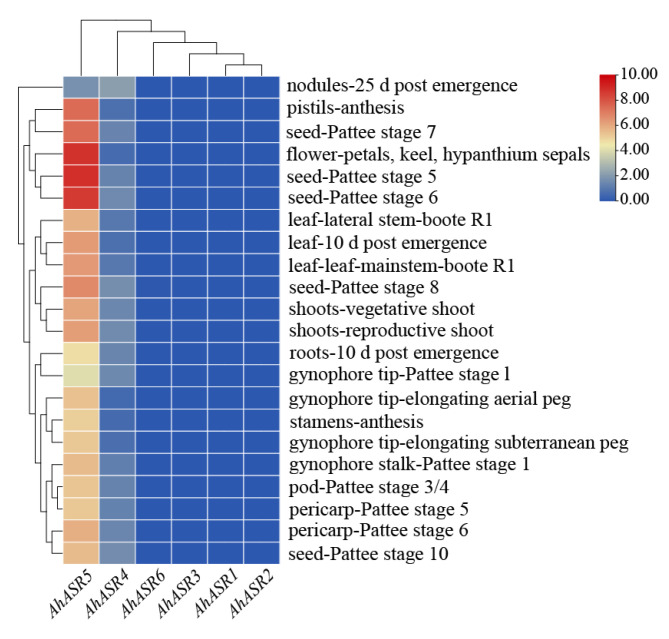
Expression analysis of *AhASR* genes in different tissues.

Transcriptome data for peanut were collected in various tissues and at different time points from the peanut-base website to analyze the expression patterns of the *ASR* gene family using log_2_ fpkm + 1. Figure 8 illustrates that *AhASR5* exhibited significant expression across multiple peanut tissues, while *AhASR4* was only detected in a limited number of tissues, such as leaves, stems, roots, flowers, seeds and pods. These two genes are likely to play crucial roles in peanut growth and development. The remaining four *ASR* genes showed no expression throughout the peanut life cycle. These results reflect that *ASR* genes may have undergone some functional differentiation in peanut treatment.

### 2.10. Expression Profiles of AhASRs in Response to PEG, NaCl, Al, and Cd

**Figure 9:**
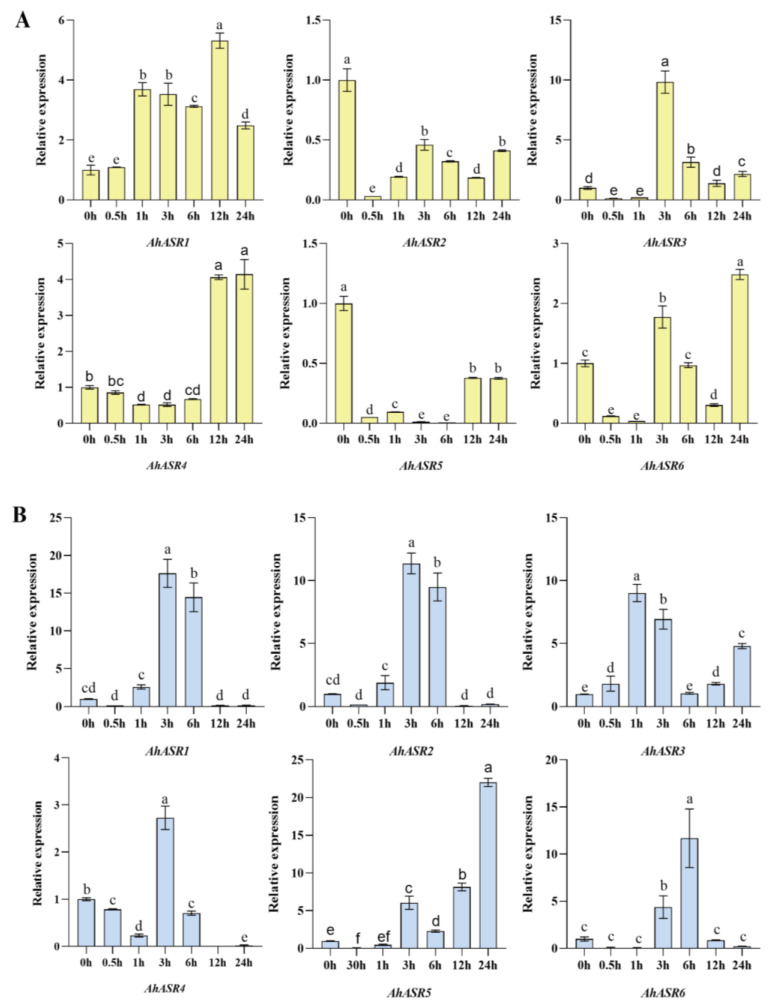
Expression levels of *AhASRs* were measured at different time points under 15% PEG stress in peanut leaves (**A**) and roots (**B**). Bars with the same letters indicate no significant difference at *p* < 0.05.

Under abiotic stress, the expression patterns of *AhASRs* in peanut leaves and roots were investigated. Under PEG treatment, the expression of *AhASR4* in leaves was upregulated at 24 h, whereas the expression of *AhASR2* and *AhASR5* was suppressed (Figure 9A). In roots, the expression levels of *AhASR1*, *AhASR2*, and *AhASR4* peaked at 3 h after PEG treatment, while *AhASR3*, *AhASR5*, and *AhASR6* reached their highest expression levels at 1, 24, and 6 h, respectively (Figure 9B).

**Figure 10:**
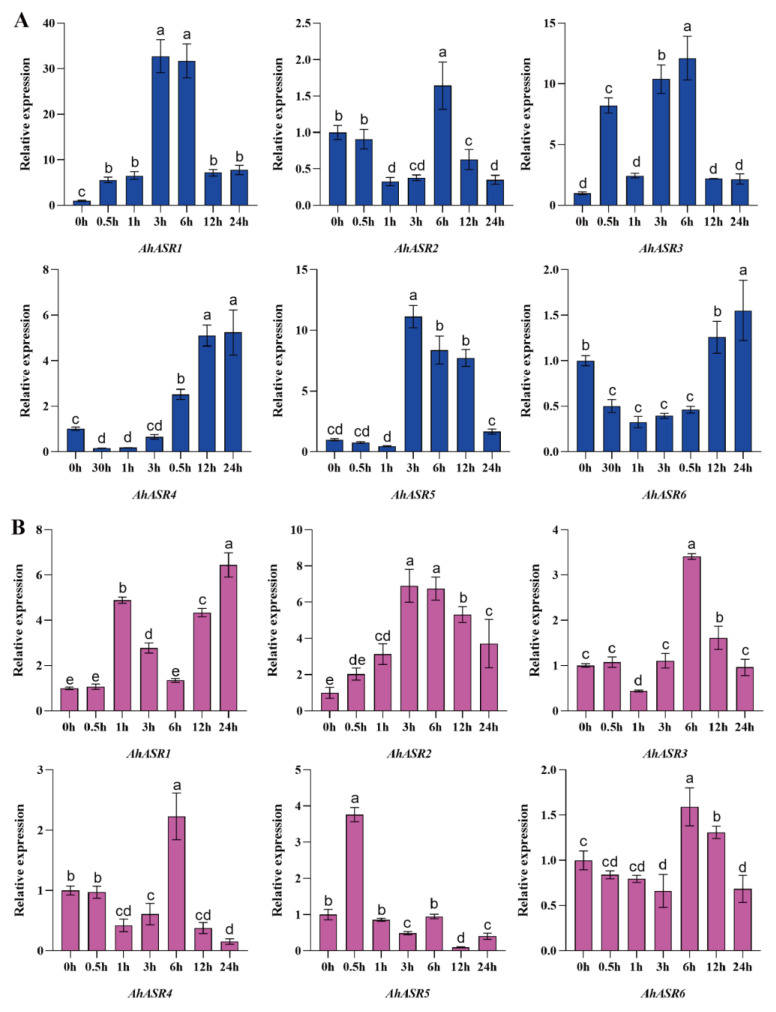
Expression levels of *AhASRs* in leaves (**A**) and roots (**B**) at different times under 200 mM NaCl stress. Bars represented by the same letters are not significantly different at *p* < 0.05.

In peanut leaves exposed to salt stress (Figure 10A), the expression levels of *AhASR1*, *AhASR3*, and *AhASR5* were significantly upregulated at 3 h. *AhASR4* showed sustained upregulation throughout the treatment, reaching levels approximately 5 times higher than that of the control. On the other hand, in peanut roots (Figure 10B), the expression levels of *AhASR3* and *AhASR4* increased significantly at 6 h of stress and then decreased at 12 and 24 h. The expression of *AhASR1* was upregulated at 24 h of stress. These results indicate that *AhASR4* in peanut leaves and *AhASR1* in roots respond to salt stress by increasing their expression, thereby improving peanut’s salt tolerance.

**Figure 11:**
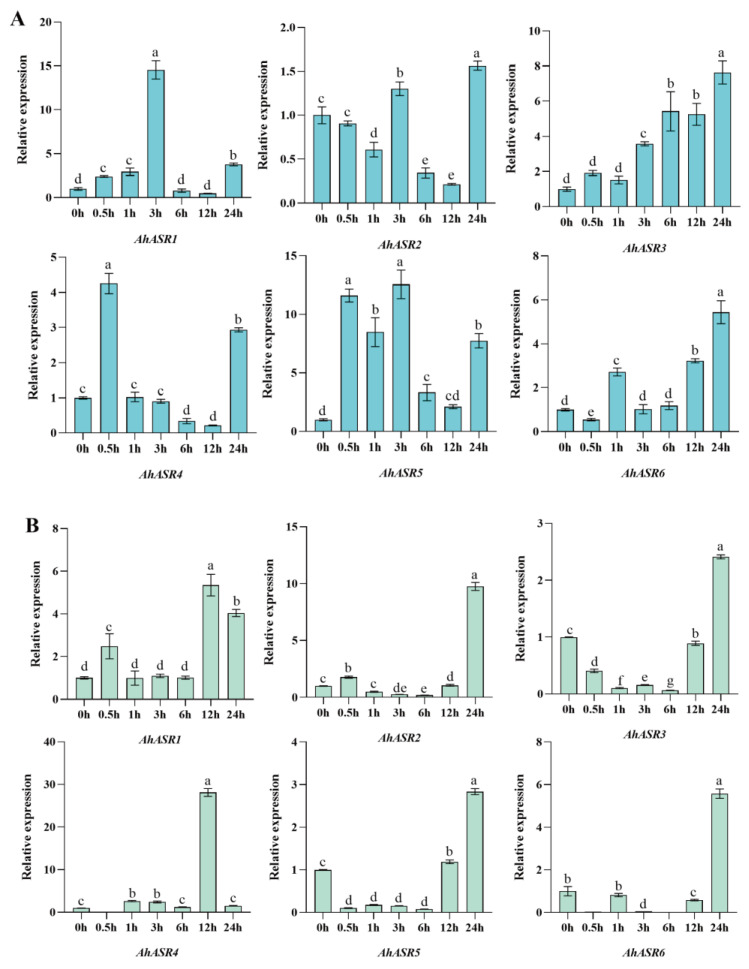
Expression levels of *AhASRs* in leaves (**A**) and roots (**B**) at different times under 0.1 mM Al stress. Bars represented by the same letters are not significantly different at *p* < 0.05.

Under Al stress, the relative expression of *AhASR1* and *AhASR5* in peanut leaves significantly increased at 3 h, followed by a decrease in expression at later time points. *AhASR3* and *AhASR6* exhibited an overall increasing trend in expression levels during the stress period, peaking at 24 h. At this time point, their expression was approximately 8 and 6 times higher than the control group, respectively (Figure 11A). In peanut roots, the expression levels of *AhASR1* and *AhASR4* significantly increased after 12 h of stress, with *AhASR4* showing the highest expression at this time point compared to the control, approximately 28 times higher. *AhASR2*, *AhASR3*, *AhASR5*, and *AhASR6* exhibited notable upregulation at 24 h of stress (Figure 11B). This indicates that the *ASR* gene family in peanut responds robustly to Al stress, particularly in the roots. These results suggest that *AhASR3* in peanut leaves and *AhASR2* in roots could be potential candidate genes for Al stress tolerance.

**Figure 12:**
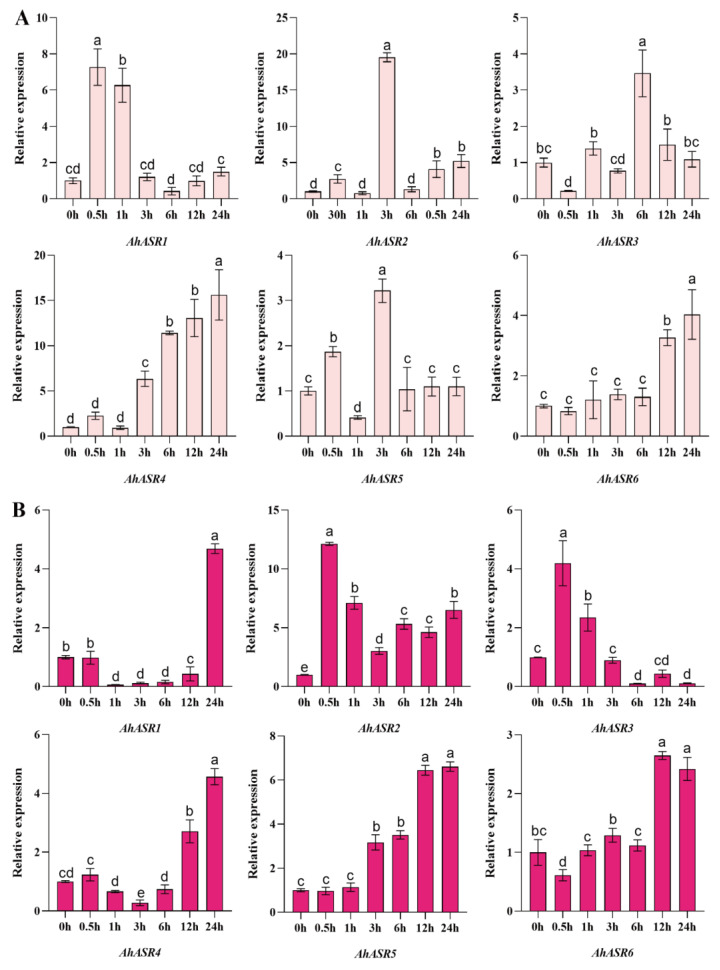
Expression levels of *AhASRs* at different times in leaves (**A**) and roots (**B**) under 2 mM Cd stress. Bars represented by the same letters are not significantly different at *p* < 0.05.

Under Cd stress, the expression of *AhASR2* in peanut leaves was highest at 3 h post-treatment and declined at subsequent times, while *AhASR4* and *AhASR6* were consistently upregulated during treatment (Figure 12A). In peanut roots (Figure 11B), *ASR* exhibited diverse expression patterns, with *AhASR3* showing continuous downregulation under stress, while *AhASR5* showed continuous upregulation in response (Figure 12B). These findings suggest that *AhASR4* in the leaf and *AhASR5* in the root of peanut are candidate genes associated with Cd stress tolerance.

## 3. Discussion

ASRs play a crucial role in regulating plant responses to various stressors, including osmotic stress, salinity, drought, and ABA [17,21,42]. This study identified six members of the peanut *ASR* gene family through genome-wide analysis. All ASR proteins exhibited conserved ABA-WDS structural domains and shared conserved motif 1, conserved motif 2, and conserved motif 3, which are believed to have specific functions (Figure 3B). The amino acid sequences of peanut ASRs were highly conserved, characterized by an ABA/WDS domain, a histidine-rich region near the N-terminus, and a lysine group-containing putative nuclear targeting signal at the C-terminus (Figure 4) similar to ASR proteins in other species [10]. While previous studies have shown that most ASRs are found in the nucleus and cytoplasm [9,43], subcellular localization predictions in the present work suggest their presence in the mitochondria and nucleus. Notably, ASR proteins present in the nucleus can function as transcription factors, regulating gene expression in response to stress [10].

The introns of the peanut *ASR* gene family became progressively shorter as evolutionary relationships developed (Figure 3C). Previous research has demonstrated that introns tend to decrease over the course of evolution [44,45]. Genes with compact structures containing fewer introns can activate genes and respond to stress-related genes efficiently [46,47]. Delayed transcripts negatively impact gene expression [48], prompting genes to adapt to various pressures and mount timely responses [49,50,51]. Specifically, in this study, it was observed that *AhASR2* and *AhASR6* had the shortest introns, potentially contributing to the heightened response of *AhASR6* to Al and Cd stress. Cis-acting elements are crucial for regulating gene expression [52]. The 2000-bp promoter region of the *AhASR* genes contains 21 cis-acting elements, including those responsive to hormones, defense mechanisms, and stress, particularly drought conditions. The widespread distribution of these elements suggests that *AhASRs* may play a significant role in regulating various biological processes. In this study, the promoter region of *AhASR4* contained binding sites for MYB involved in drought and light, anaerobic response elements, and cis-acting elements responsive to MeJA, GA, and ABA. The promoter region of *AhASR5* demonstrated photoresponsiveness, circadian rhythm regulation, anaerobic induction, palisade tissue cell differentiation, endosperm expression, and salicylic acid and MeJA hormone responsive cis-acting elements. In *Tetragonia tetragonides* (Pall.) Kuntze, the *TtASR* promoter region contains abundant cis-acting elements, including ABRE and MYB transcription factor regulatory sites. These stress-related promoters enable the *TtASR* to play crucial roles in resistance to drought and salt–alkali conditions [53]. Similarly, the MYB and ABRE cis-acting elements of *OsASR1* in rice enhance its drought resistance [54]. Furthermore, introducing the *ASR* promoter, which contains MeJA-responsive elements, into rice significantly improves its resistance to salt and drought [55]. Previous studies have demonstrated that ABA and MeJA signaling pathways can induce stomatal closure and modulate the expression of associated genes in plants experiencing abiotic stress [56,57]. MYB transcription factors bind to the promoter regions of genes and collaborate synergistically with other regulatory factors to control the expression of *ASR* genes under varying environmental conditions [58]. The expression of *AhASR4* in this study was consistently upregulated under Cd, drought, and salt stress conditions, whereas *AhASR5* was significantly upregulated under drought, Al, and Cd stress. This suggests that *AhASR4* and *AhASR5* may be involved in hormonal responses and play crucial roles in mitigating stress and adversity. Therefore, we hypothesize that stress resistance in peanut may be enhanced through the regulation of cis-acting elements, such as ABA, MeJA, and MYB, in the promoter region, thereby improving the plant’s overall stress resilience. The growth and development expression heatmap of peanut displayed high gene expression levels of *AhASR5* across all tissues and developmental stages of peanut, suggesting its importance in peanut growth, development, and resistance to abiotic stress (Figure 8).

Gene replication plays a crucial role in expanding gene families and developing new functions, enhancing the adaptability and diversity of organisms in response to environmental changes [59,60,61]. Tandem duplication and fragment duplication are key mechanisms behind the emergence and evolution of gene families [62,63]. In this study, two instances of whole genome replication were identified, involving the *AhASR1* and *AhASR4* as well as *AhASR2* and *AhASR6* pairs, suggesting that the *ASR* gene family in peanut originated from whole genome replication. Notably, *AhASR4* and *AhASR5* were closely linked on Chr15, with similar gene and protein structures and expression patterns across various peanut tissues, indicating a tandem duplication event between these two genes. These findings imply that the formation of *AhASRs* may be attributed to replication events and tandem duplication, shedding light on the evolution of *AhASRs* [64,65]. Furthermore, the degree of genomic collinearity between species reflects their evolutionary relationship [66], with higher homology observed between peanut *ASR* genes and soybean *ASR* genes compared to alfalfa based on collinearity analysis (Figure 6B). The Ka/Ks ratio, which compares nonsynonymous to synonymous substitutions, serves as a crucial metric for evaluating gene evolution rates and the associated selection pressures. A higher Ka/Ks ratio may indicate that the gene has undergone substantial functional changes during evolution, whereas a lower ratio suggests that the gene has maintained its functions over time [67]. An analysis of the Ka/Ks values revealed that the *ASR* gene in peanut experienced purifying selection throughout its evolutionary development (Table S3). This indicates that the *ASR* gene shares a common ancestral lineage and has maintained a high degree of conservation throughout evolutionary history. Furthermore, the analysis of the interaction network (Figure 7) revealed interactions between the peanut ASR protein and a protein containing a C2H2-type domain as well as two members of the peroxiredoxin family. We propose that these interacting proteins may perform analogous functions, potentially contributing collectively to the detoxification of peroxides and enhancing cellular resistance to oxidative stress.

ASR proteins are known to play a crucial role in enhancing abiotic stress tolerance in plants. For instance, the overexpression of *TtASR* in *Tetragonia tetragonides* has been shown to confer resistance to salt, osmotic stress, and ABA treatment [68]. Similarly, the overexpression of *BdASR4* in *Brachypodium distachyon* has been found to increase drought tolerance in transgenic crops [69]. Moreover, under drought stress conditions, *PhyASR2* from *Phyllostachys pubescens* triggers the overexpression of ROS-related genes in transgenic rice [70]. ASR proteins help improve plant osmotic balance and the antioxidant system by regulating ROS homeostasis, thereby reducing oxidative damage in various plants, such as maize and lychee. This ultimately leads to enhanced drought resistance by minimizing water loss, cell damage, and accumulation of ROS [71,72]. *OsASR1* overexpression in transgenic rice plants enhances ABA accumulation by regulating osmotic pressure and stomatal closure expression to improve tolerance [29]. Furthermore, *OsASR5* in rice binds to cis elements in the promoter of Al- responsive genes to regulate gene expression [26]. This research found that the genes *AhASR4* and *AhASR5* were expressed in all tissues and developmental stages of peanut growth, indicating their important role in peanut development (Figure 8). The qRT-PCR analysis results showed that (Figure 9, Figure 10, Figure 11 and Figure 12) *AhASR4* expression was consistently increased in response to heavy metal, drought, and salt stress, while *AhASR5* expression significantly increased specifically under drought, Al, and Cd stress conditions. Therefore, we propose that the *ASR* gene in peanut may improve resistance to abiotic stress. The results of qRT-PCR analysis showed (Figure 9, Figure 10, Figure 11 and Figure 12) that the expression of *AhASR4* continued to increase under drought, salt, and Cd stress, while the expression of *AhASR5* significantly increased under drought, Al, and Cd stress. Therefore, we believe that peanut *ASR* genes may improve peanut resistance to abiotic stress, especially in response to abiotic stressors, such as drought, salinity, and heavy metals. Genetic engineering technology can enhance the expression of *ASR* genes in peanut, thereby enhancing peanut’s tolerance to abiotic stressors, such as drought, salinity, and heavy metals [73].

Collectively, these studies underscore the distinct mechanisms through which members of the peanut *ASR* family respond to various stressors. These mechanisms include the upregulation of gene expression, the regulation of biological processes, and the binding to cis-acting elements in promoters. Consequently, our findings establish a foundation for further research into the role of the *ASR* gene family in abiotic stress responses, and they serve as a reference for efforts aimed at developing stress-resistant peanut varieties. Nevertheless, the functions of these genes require further elucidation in future studies, such as overexpression, knockout, and transgenesis techniques utilizing the CRISPR/Cas system.

## 4. Materials and Methods

### 4.1. Identification and Sequence Analysis of AhASR Gene Families

The whole genome and annotation information files of peanut were downloaded from PeanutBase [74] (https://www.peanutbase.org/, accessed on 4 November 2023). Potential members of AhASRs were obtained by BLAST. Functional annotations were filtered for Protein family database (Pfam) identifiers of the ABA-WDS domain using the online websites of Pfam (PF02496) (http://pfam.xfam.org/, accessed on 5 November 2023). InterPro (https://www.ebi.ac.uk/interpro/, accessed on 5 November 2023), and the NCBI Conserved Domain Database (CDD) (https://www.ncbi.nlm.nih.gov/cdd, accessed on 5 November 2023). Sequences lacking the conserved structural domains were excluded to pinpoint members of the AhASR family. Protein physicochemical properties were assessed using ExPASy-ProtParam (https://web.expasy.org/protparam/, accessed on 6 November 2023) and SOPMA (https://smart.embl.de/, accessed on 6 November 2023) for analyzing the protein structure of AhASRs. SWISS-MODEL (https://www.swissmodel.expasy.org/, accessed on 6 November 2023) was utilized to predict the high-level structure of AhASR proteins. Subcellular localization of AhASR proteins was predicted with WoLF PSORT II (https://wolfpsort.hgc.jp/, accessed on 6 November 2023).

### 4.2. Analysis of the Characteristics of AhASR Genes

Gene density information was acquired, and chromosome distribution was visualized using the software TBtools (v 2.119). The full-length amino acid sequences of ASR proteins from soybean, cowpea, chickpea, kidney bean, *Medicago truncatula*, and rice were retrieved from Phytozome v.13 (https://phytozome.jgi.doe.gov/pz/portal.html, accessed on 16 November 2023). These protein sequences of the identified ASRs were utilized to generate a phylogenetic evolutionary tree through the maximum likelihood method in MEGA7.0 software with the parameter bootstrap method set to 1000.

The conserved motif composition of AhASR members (motif search limit 10) was analyzed using the online site MEME (http://meme-suite.org/tools/meme, accessed on 7 November 2023), and the structural domains of AhASR members were analyzed via the NCBI (https://www.ncbi.nlm.nih.gov/Structure/cdd/wrpsb.cgi, accessed on 7 November 2023) online site to analyze the structural domains of members of AhASRs.

DNAMAN 5.2.2 was used to compare the sequences of AhASRs proteins, and then the amino acid frequency analysis of AhASRs proteins was performed through the online website Weblogo (http://weblogo.threeplusone.com/, accessed on 7 November 2023).

The 2000-bp nucleotide sequence upstream of the transcription start site of the *AhASR* gene was submitted to PlantCARE for cis-acting element prediction.

Based on the BLAST results and gene localization information, collinear gene pairs were identified using MCScanX in TBtools (v 2.119), and the data were plotted using Circos. The software TBtools (v 2.119) was used to calculate the Ka/Ks ratio for each gene pair. The Ks value was subsequently converted into divergence time, expressed in millions of years, and calculated as described [75].

The STRING database (https://string-db.org, accessed on 23 August 2024) was used to predict protein–protein interaction networks for six AhASR protein sequences [76].

### 4.3. Tissue Expression Analysis of AhASR Genes

RNA-seq data for 22 peanut tissues were obtained from the PeanutBase database (https://peanutbase.org/, accessed on 20 November 2023). TBtools (v 2.119) software was used to draw expression heat maps.

### 4.4. Plant Cultivation, RNA Extraction, and qRT-PCR Analysis of AhASR Genes

Peanut variety Huayu 23 was used as the plant material. Peanut seedlings were cultivated using hydroponics, with the seeds being surface sterilized and placed in Petri dishes for germination. The water in the hydroponic system was changed daily. Once the seeds had sprouted, 6 peanut seeds were transplanted into the hydroponic box. The greenhouse conditions were maintained at 24 °C with a photoperiod of 14 h of light and 10 h of darkness in order to cultivate peanut to the 4-leaf stage.

At the 4-leaf stage, peanut seedlings with uniform growth were subjected to drought stress treatment using 20% PEG 6000. The treatment group received 1L 1/2 Hoagland solution with 20% PEG 6000, while the control group received 1L 1/2 Hoagland solution. Additionally, other stress treatments included 200 mM NaCl for salt stress, 0.1 mM AlCl_3_ for Al stress, and 2 mM CdCl_2_ for Cd stress, following a similar procedure as described for the drought stress treatment. Peanut roots and leaves were collected at various time points (0 h, 0.5 h, 1 h, 3 h, 6 h, 12 h, and 24 h) and stored for RNA extraction after freezing in liquid nitrogen and storing at −80 °C.

Based on the coding sequences of *AhASRs*, qRT-PCR primers (Table S2) were designed using the online software NCBI (https://www.ncbi.nlm.nih.gov/, accessed on 23 November 2023). Total RNA was extracted from peanut leaves and roots using Trizol reagent (CwBio, Beijing, China) and its quality was assessed using 1% agarose gel electrophoresis. RNA was reverse-transcribed into cDNA (Thermo Fisher Scientific, Waltham, MA, USA). The qRT-PCR assay was performed using TaLent Fluorescence Quantification Kit (Tiangen, Beijing, China) and calculated using 2^−ΔΔCT^ with *actin* from peanut serving as an internal reference [77,78].

### 4.5. Statistical Analysis

The data were analyzed using SPSS statistics software (version 27, IBM, Chicago, IL, USA). After confirming the normality and homogeneity of the data, variance analysis was performed on each group. All experimental results were expressed as the standard deviation (SD) (*n* = 3). A minimal significant difference test (*p* < 0.05) was conducted a priori to compare differences between different methods.

## 5. Conclusions

In this study, six *ASR* genes were identified in peanut. These *AhASR* genes were found to be unevenly distributed across four chromosomes of the peanut genome. Structural analysis of the genes revealed that the *AhASRs* are highly conserved, exhibiting extensive similarity among the peanut *ASR* members. Our findings suggest that *AhASR4* in peanut leaves may serve as a potential candidate gene for tolerance to drought, NaCl, and Cd stress. Additionally, *AhASR5* in peanut roots demonstrated tolerance to drought and Cd. Moreover, *AhASR3* in peanut leaves and *AhASR2* in roots exhibited tolerance to the heavy metal Al. Overall, this study provides a comprehensive examination of the characterization of peanut *ASR* genes, offering valuable insights for further exploration into the functions and molecular mechanisms of *AhASR* genes in response to abiotic stress.

## Figures and Tables

**Table 1:** Identification of *ASR* genes in peanut.

Sequence ID	Exon Number	Open Reading Frame Length (bp)	Number of Amino Acid	Molecular Weight (kDa)	Theoretical pI	Unstable Parameters	Fat Factor	Total Mean Hydrophilic Value	Subcellular Localization
*AhASR1*	2	356	117	13.67	6.98	66.48	56.84	−1.168	mitochondrion
*AhASR2*	2	636	211	22.76	5.34	49.67	25.12	−1.508	nucleus
*AhASR3*	2	345	114	13.18	6.24	47.83	47.28	−1.427	mitochondrion
*AhASR4*	2	354	117	13.38	6.16	38.63	46.92	−1.338	mitochondrion
*AhASR5*	2	354	117	13.4	6.55	35.79	51.2	−1.242	mitochondrion
*AhASR6*	2	672	223	23.96	5.35	49.87	23.77	−1.501	nucleus

## Data Availability

Data are contained within the article or Supplementary Materials.

## Supplementary Materials

The following supporting information can be downloaded at: https://www.mdpi.com/article/10.3390/ijms252011008/s1.
